# Neonatal CNS Human Parechovirus Infections in Western Pennsylvania in the 2024 Season

**DOI:** 10.1002/jmv.70870

**Published:** 2026-03-11

**Authors:** Gal Yovel, Jessica Elizabeth Packard, Justin C. Wang, Sarah Maya, Ethan Chi, Sara Walters, Megan Culler Freeman

**Affiliations:** ^1^ Department of Pediatrics University of Pittsburgh School of Medicine Pittsburgh Pennsylvania USA; ^2^ University of Pittsburgh School of Medicine Pittsburgh Pennsylvania USA

**Keywords:** cerebrospinal fluid, enterovirus, febrile neonates, human parechovirus, meningoencephalitis

## Abstract

Human Parechovirus A (PeV‐A) is a virus with near‐universal infection by age five; however, neonatal infections can lead to meningoencephalitis, sepsis, and death. Prior to the COVID‐19 pandemic, PeV‐A showed biennial seasonality with late summer peaks, but multiple viruses have had shifted circulation post‐pandemic. PeV‐A is not universally included in neonatal sepsis testing; thus, the frequency and clinical spectrum of PeV‐A neonatal meningoencephalitis are not fully described. We sought to evaluate the epidemiology, seasonality, and clinical presentation of neonatal PeV‐A in the 2024 season. We collected remnant cerebrospinal fluid samples from febrile infants under 60 days at a single children's hospital in Southwestern Pennsylvania and assessed for PeV‐A and enterovirus (EV). Six out of 107 (5.6%) febrile infants were positive for PeV‐A and 24 (22.4%) were positive for EV. PeV‐A infections occurred from June to September. PeV‐A positive patients had a distinct combination of higher maximum temperature, rash, and leukopenia without pleocytosis. None of these infants had severe disease. Systematic surveillance of PeV‐A is required to completely understand ongoing PeV‐A circulation patterns, expected clinical course, and long‐term developmental implications.

**Abbreviations**
CDC: Centers for Disease Control and Prevention
CNS: central nervous system
CSF: cerebrospinal fluid
EEG: electroencephalogram
EV: enterovirus
MRI: magnetic resonance imaging
PeV‐A: human parechovirus
RBC: red blood cell
WBC: white blood cell

## 1 Introduction

Human Parechovirus (PeV‐A) is a non‐enveloped, single‐stranded, positive‐sense RNA virus belonging to the *Picornaviridae* family, which also includes enteroviruses (EV) and rhinoviruses [1]. Transmission of PeV‐A occurs primarily through fecal‐oral and respiratory routes, leading to near‐universal infection by 5 years of age. While most infections are asymptomatic or cause only mild symptoms, PeV‐A can cause severe illness in young infants, including neonatal sepsis, meningoencephalitis, seizures, and death [1, 2, 3, 4, 5]. Nineteen genotypes have been identified, with PeV‐A type 3 (PeV‐A3) most often implicated in CNS disease [6, 7]. National data on PeV‐A incidence, circulation patterns, and rates of severe infection are limited due to a lack of systematic surveillance and routine testing.

Prior to the COVID‐19 pandemic, studies in Europe and the US showed biennial circulation for PeV‐A, with infections typically peaking in late summer [8, 9, 10]. Like many viruses, PeV‐A circulation was disrupted during the COVID‐19 pandemic due to non‐specific interventions, such as masking and social distancing [11, 12]. Cases returned in 2022, with pre‐pandemic infection rates reported in the US; however, circulation patterns have yet to be reestablished [8].

Febrile infants within the first 60 days of life are evaluated to rule out severe bacterial infections, including with a lumbar puncture to assess for meningitis. Most cases are attributed to viral pathogens; however, PeV‐A testing is not universally included, contributing to limited epidemiologic data and an incomplete understanding of the clinical presentation of PeV‐A in neonates. Due to under recognition and under testing, the effects on long‐term neurodevelopmental outcomes of these infants are also understudied. Patients with PeV‐A neonatal CNS disease can have neurodevelopmental impairment [13, 14].

To better understand the prevalence and presentation of PeV‐A in neonatal sepsis in Western Pennsylvania, we conducted a retrospective study of infants under 60 days of age who presented to the UPMC Children's Hospital of Pittsburgh between April and December 2024 with neonatal sepsis. We collected remnant cerebrospinal fluid (CSF) and conducted research lab testing for PeV‐A. Clinical course and laboratory values, including EV test results, were obtained via chart review. We characterized the clinical presentation of CNS infections with PeV‐A or EV as compared to febrile control patients without PeV‐A or EV.

## 2 Materials and Methods

### 2.1 Study Criteria

Ethical approval was granted by the Institutional Review Board at the University of Pittsburgh (IRB# STUDY24010106*)* for this retrospective study involving residual specimens. Infants aged 60 days or younger who presented to the UPMC Children's Hospital of Pittsburgh in Pittsburgh, Pennsylvania, USA between April 1 and December 31, 2024 and had remnant CSF available after laboratory testing were screened for inclusion. Patients were eligible if they were hypothermic (≤36.0°C) or febrile (≥38.0°C) by history or chart review and if they presented from the community without significant prior medical history. Patients who were culture positive for bacterial infections or had bacterial infections diagnosed by their treating physician, such as acute otitis media, pneumonia, or skin and soft tissue infection, were excluded. Remnant CSF was tested for PeV‐A in the research laboratory. Patients were considered positive for EV via chart review of clinical testing if they were EV positive from CSF or from blood in combination with pleocytosis. Patients negative for PeV‐A and EV in the CSF were considered “febrile controls.” None of the hypothermic infants in our cohort were PeV‐A positive and were excluded from further analysis.

### 2.2 Rna Extraction

CSF samples were spun for 10 min at 1500 × *g*. RNA was extracted from CSF using the Qiagen QIAMP Viral RNA Minikit (Qiagen, 52904) according to the manufacturer's protocol with the addition of a 1‐min centrifugation at 20,000 × *g* after the addition of buffer AW2. RNA extracts were then transferred to de‐identified, study‐specific labeled tubes and stored at −80°C.

### 2.3 Viral Testing

For PeV‐A conducted in the research laboratory, RT‐qPCR was conducted using the Ambion AgPath ID One‐Step RT‐PCR Kit with a Quant Studio 5 instrument (Applied Biosystems). AN345 and AN344 primers with AN257 probe were used as previously described [15]. Parechovirus A Type 3 2012 (BEI US/MO‐KC/2012/006F) at 1:1000 dilution was used for a positive control. For EV testing conducted in the clinical laboratory, specimens were extracted using the EasyMag (bioMérieux). EV detection was completed with Elitech ASR reagents on the ABI 7500 Real‐Time PCR System (Applied Biosystems).

### 2.4 Study Definitions

We defined CSF pleocytosis as more than 15 WBC per microliter (μL) in infants under 28 days and more than 9 WBC/μL in infants between 29 and 60 days. Leukopenia was defined as a peripheral WBC count <5 × 109 cells/L. A traumatic lumbar puncture was defined as red blood cell (RBC) count >1000 RBC/μL.

### 2.5 Statistical Analysis

Continuous variables were summarized using median and interquartile ranges (IQR), and categorical variables as frequencies and percentages (%). Normality of continuous variables was assessed using the Shapiro‐Wilk test. When approximate normality was satisfied, comparisons of continuous variables were performed using Welch's two‐sample *t*‐test; otherwise, the Wilcoxon rank‐sum test was used. Comparisons of categorical variables were conducted using the chi‐squared test when all expected cell counts were at least 5; otherwise, Fisher's exact test was applied. Pairwise comparisons were conducted for each variable, and multiple testing was addressed using the Benjamini–Hochberg procedure. Adjusted *p*‐values are reported, and values <0.05 were considered statistically significant. All analyses were performed using R Version 4.4.1.

## 3 Results

### 3.1 Clinical Characteristics of PeV‐A and EV Positive Febrile Infants

**FIGURE 1:**
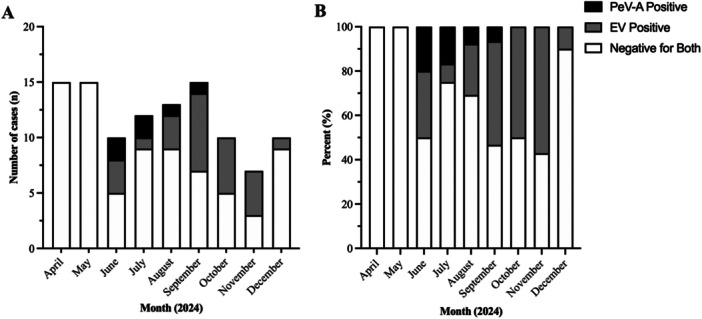
Distribution of Human Parechovirus and Enterovirus cases in 2024. Distribution of dates of presentation by month, with PeV‐A positive samples represented in black (*n* = 6), EV positive samples represented in gray (*n* = 24), and samples negative for both represented in white (*n* = 77). A. Depicts total number and B. Depicts percentage of cases.

Out of 107 infants that met the inclusion criteria, 6 (5.6%) were positive for PeV‐A and 24 (22.4%) were positive for EV. Of the EV‐positive patients, 22 were positive in CSF, one was positive in both blood and CSF, and one had EV‐positive blood in the absence of CSF testing. One of the EV‐positive patients had a coincident bacterial urinary tract infection and was excluded from the analysis of laboratory and clinical data. Of the remaining patients, 17 (73.9%) of EV‐positive patients and 3 (50%) of PeV‐A positive patients had clinical respiratory viral testing sent. Three (17.6% of tested) EV‐positive patients were positive for the rhinovirus/enterovirus target, but no other respiratory viruses were detected in patients in the EV or PeV‐A group. The remaining 77 CSF samples were negative for both PeV‐A and EV and comprised the febrile control group. PeV‐A positive samples were identified from June to September (Figure 1). EV positive samples were identified from June through December (Figure 1).

### 3.2 Clinical Presentation and Lab Values of Infants Infected With PeV‐A or EV

Data comparing demographics and clinical features of PeV‐A positive, EV positive, and febrile control infants were analyzed (Table 1). The median age of PeV‐A positive infants (16 days) was not statistically different from EV positive patients (23 days) or febrile controls (30 days). Male infants were 66.7% of the PeV‐A‐positive patients and 47.8% of the EV‐positive patients and 53.2% of the febrile controls. Older siblings were present in the homes of 83.3%, 87%, and 72.7% of PeV‐A positive, EV positive, and febrile control infants, respectively.

**TABLE 1:** Demographics and clinical presentation.

	PeV‐A (*n* = 6)	EV (*n* = 23)†	Negative for both (*n* = 77)	Adjusted *p‐*value (PeV‐A vs. EV)	Adjusted *p*‐value (PeV‐A vs. Negative for both)	Adjusted *p‐*value (EV vs. Negative for both)
**Age (days), median (IQR)**	16, (10.5–23)	23, (13–34.5)	31, (18‐46)	0.79	0.35	0.56
**Sex, Male (%)**	5, (66.7)	11, (47.8)	41, (53.2)	> 0.99	> 0.99	> 0.99
**Older sibling at home (%)**	5, (83.3)	20 (87)	56 (72.7)	> 0.99	> 0.99	0.53
**Days hospitalized, median (IQR)**	1.5, (0.25–2)	1, (1–2)	1, (1–2)	> 0.99	> 0.99	> 0.99
**Max Temp (°C), median (IQR)**	38.75, (38.53–38.9)	38.4, (38.25–39.1)	38.1, (37.8–38.6)	> 0.99	0.04*	0.04*
**Rash (%)**	5, (83.33)	4, (17.4)	13, (16.9)	0.04*	0.03*	> 0.99
**Diarrhea (%)**	0, (0)	0, (0)	3, (3.9)	> 0.99	> 0.99	> 0.99
**Vomiting (%)**	0, (0)	1, (4.3)	4, (5.2)	> 0.99	> 0.99	> 0.99
**Cough (%)**	0, (0)	0, (0)	17, (22.1)	> 0.99	0.78	0.06
**Congestion (%)**	1, (16.7)	1, (4.3)	30, (39)	0.84	0.87	0.04*
**Rhinorrhea (%)**	0, (0)	0, (0)	2, (2.6)	> 0.99	> 0.99	> 0.99
**Fussiness (%)**	5, (83.33)	16, (69.6)	30, (39)	> 0.99	0.32	0.06
**Increased work of breathing (%)**	0, (0)	2, (8.7)	10, (13)	> 0.99	> 0.99	> 0.99

†These results were obtained from 24 patients who were positive for EV (excluding 1 patient with bacterial infection).

*Results indicate statistical significance after Benjamini–Hochberg correction.

PeV‐A‐positive infants were hospitalized for a similar duration as EV‐positive infants and febrile controls, with a mean of 1–1.5 days. PeV‐A and EV‐positive infants had significantly higher temperatures than febrile controls, with medians of 38.8°C, 38.4°C, and 38.1°C, respectively. As for clinical presentation, PeV‐A positive infants more frequently presented with rash compared to EV positive or febrile control infants. Although not statistically significant, PeV‐A‐positive infants had a trend toward increased fussiness. No other clinical presentation features were significantly different between PeV‐A‐positive and EV‐positive infants.

We assessed the laboratory features of each condition (Table 2). PeV‐A positive infants had significantly lower peripheral WBC counts (4.9 × 10⁹ cells/L) compared to febrile control infants (9.95 × 10⁹ cells/L). There were no statistically significant differences in procalcitonin levels or blood platelet levels between groups. PeV‐A positive infants had significantly lower CSF WBC (2 cells/μL) compared to EV positive infants (10 cells/μL). Other CSF parameters were similar between PeV‐A and EV‐positive groups. There were no significant differences in the receipt of antibiotics across patients of any group. While two EV‐positive infants underwent electroencephalogram (EEG) and one received magnetic resonance imaging (MRI), none of the PeV‐A‐positive infants in our study had clinical seizures or were evaluated with EEG or MRI.

**TABLE 2:** Clinical presentation.

	PeV‐A (*n* = 6)	EV (*n* = 23)†	Negative for both (*n* = 77)	*p*‐value; PeV‐A vs. EV	*p*‐value; PeV‐A vs. negative for both	*p*‐value; EV vs. negative for both
**Procalcitonin (ng/ml), median (IQR)**	0.18, (0.11–0.24)	0.19, (0.11–0.31)	0.17, (0.09–0.55)	> 0.99	> 0.99	> 0.99
**Blood WBC (× 10⁹ cells/L), median (IQR)**	4.9, (3.57–6.83)	7.88, (6.68–11.08)	9.95, (7.43–13.23)	0.10	0.04*	0.40
**Blood Platelet (× 10⁹ cells/L), median (IQR)**	364, (272–373)	336, (267–402)	383, (264–494)	> 0.99	> 0.99	0.63
**CSF WBC (/μL), median (IQR) ††**	2, (0–2)	10, (3.75–73.75)	2, (0–5)	0.05*	0.63	0.01*
**CSF RBC (/μL), median (IQR) ††**	0, (0–7)	11.5, (5.75–120)	1.5, (0–11.5)	0.23	0.81	0.04*
**CSF Protein (mg/dl), median (IQR) ††**	54, (54–69)	68.5, (54–79.5)	54, (43.5–65)	0.40	0.84	0.04*
**CSF Glucose (mg/dl), median (IQR) ††**	56, (51–66)	47, (40.75–53.25)	53, (48–58)	0.22	0.69	0.08
**Received Antibiotics (%)**	6, (100)	21, (91.3)	75, (97.4)	> 0.99	> 0.99	0.64
**MRI (%)**	0, (0)	1, (4.3)	3, (4.1)	> 0.99	> 0.99	> 0.99
**EEG (%)**	0, (0)	2, (8.7)	3, (3.9)	> 0.99	> 0.99	> 0.99

† These results were obtained from 24 patients who were positive for EV (excluding 1 patient with bacterial infection) †† Data analysis excluded samples with a traumatic lumbar puncture of RBC > 1000 RBC/μL * Results indicate statistical significance after Benjamini–Hochberg correction.

## 4 Discussion

In our study, we found that 5.6% of remnant CSF from febrile neonates were positive for PeV‐A. This is similar to prior reports of 7% positivity in the Midwestern United States [16], but higher than a California study with only 2.5% PeV‐A positivity [17]. These differences may be attributed to several factors, including sampling dates, differences in sample size, or geographic variation, as viral transmission may vary with climate, humidity, and temperature. Together, PeV‐A and EV accounted for over 25% of neonatal sepsis presentations attributable to viral infection in the summer and autumn seasons at our center. We also noted a shift in seasonality of PeV‐A circulation toward the early summer months in the 2024 season; however, we do not have local data with which to compare, so it is unknown if these local circulation patterns are due to geography or a changing post‐pandemic landscape [11, 18, 19].

Fever, rash, and fussiness have previously been described as the symptomatic triad of PeV‐A infections [4, 16, 20]. Data from our study supported this with significantly higher fever and rash in PeV‐A patients. Increased fussiness did not meet the significance threshold, perhaps due to the low total number of PeV‐A positives. Cough and congestion were highest in the febrile control group, perhaps shifting PeV‐A and EV lower on the differential diagnosis, though these symptoms are insufficient to change existing neonatal sepsis testing algorithms.

PeV‐A is associated with severe CNS disease in the literature, and as such, patients with identified PeV‐A will often undergo additional diagnostics to assess for neurological abnormalities. Independent multistate studies of infants with PeV‐A in the 2022 season reported that 27–30% of patients had complicated disease [14, 19]. As this was the first season after the pandemic related hiatus of PeV‐A circulation, it is unclear if the percentage of complicated disease may be elevated related to waning of pre‐existing maternal antibody protection in this cohort or if other factors such as shifting genotype dynamics may factor into severity. Our data, while limited in number, included only mild cases of neonatal meningoencephalitis. Because our PeV‐A testing was performed on remnant specimens, clinicians did not know that patients were positive when assessing the need for brain imaging or seizure monitoring. None of the infants in our study with PeV‐A CNS disease had MRIs or EEGs performed based on their clinical presentation and none required ICU‐level care. While multiplex molecular PCR panels exist and are in use at many institutions, PeV‐A is not a pathogen with universal screening in neonatal sepsis. As patients with severe disease are more likely to be tested for PeV‐A, it is unknown what proportion of PeV‐A‐infected infants may have long‐term neurodevelopmental deficits, thus making it challenging for physicians to provide appropriate anticipatory guidance.

The limitations of our study are mostly due to its retrospective design. Since infants were treated by different clinicians, clinical interpretation and documentation of presenting symptoms may have varied, potentially impacting consistency of reported data, particularly with subjective findings such as “fussiness.” We were limited to remnant CSF for research laboratory PeV‐A testing, meaning if CSF was not collected or if the entire volume was used for clinical laboratory testing, those patients were not included in the study. Serum diagnostics for PeV‐A were not available at our center during the study period.

While our study may be limited by the small number of PeV‐A positive cases over a single season, it highlights that larger, multi‐center and multi‐season studies are required to better capture the clinical spectrum of PeV‐A infection in young infants and that the severity of disease may change over subsequent circulation seasons. Additional studies, including prospective surveillance of other specimen types, such as respiratory or stool, are needed to better define seasonality, duration of shedding, and genotype‐associated clinical presentation.

## Data Availability

The data that support the findings of this study are available on request from the corresponding author. The data are not publicly available due to privacy or ethical restrictions.
